# A needs assessment on addressing environmental health issues within reproductive health service provision: Considerations for continuing education and support

**Published:** 2017-12-15

**Authors:** Linzi Williamson, Sarah Sangster, Melanie Bayly, Kirstian Gibson, Karen Lawson, Megan Clark

**Affiliations:** 1 Deparment of Psychology, University of Saskatchewan, Saskatchewan, Canada; 2 Saskatchewan Prevention Institute, Saskatchewan, Canada

## Abstract

**Background:**

This needs assessment was initially undertaken to explore the beliefs and knowledge of nurses and physicians about the impact of environmental toxicants on maternal and infant health, as well as to describe current practice and needs related to addressing environmental health issues (EHI).

**Methods:**

One hundred and thirty-five nurses (n = 99) and physicians (n = 36) working in Saskatchewan completed an online survey. Survey questions were designed to determine how physicians and nurses think about and incorporate environmental health issues into their practice and means of increasing their capacity to do so.

**Results:**

Although participants considered it important to address EHIs with patients, in actual practice they do so with only moderate frequency. Participants reported low levels of knowledge about EHIs’ impact on health, and low levels of confidence discussing them with patients. Participants requested additional information on EHIs, especially in the form of online resources.

**Conclusion:**

The results suggests that while nurses and physicians consider EHIs important to address with patients, more education, support, and resources would increase their capacity to do so effectively. Based on the findings, considerations and recommendations for continuing education in this area have been provided.

## Introduction

Exposure to environmental toxicants prenatally or in infancy is a risk factor for a number of conditions including ADHD, cognitive delays, asthma, diabetes and cancer.^1^–^8^ Numerous researchers and health organizations [e.g., Society of *Obstetricians and Gynaecologists* of Canada (SOGC)] have called for timely action to identify and reduce exposure to toxic environmental agents for pre- and post-natal women.^9^–^14^ Care providers are in an ideal position to educate potential parents about environmental health risks and protective actions in the prenatal and even preconception care periods.^15^–^18^ However, evidence suggests there may be little discussion of environmental health issues (EHIs) within the reproductive health care context.^19^–^21^ While many health care providers are in favour of discussing EHIs in the context of maternal and infant health,^23^–^29^ some have noted their low knowledge levels,^21^,^27^–^30^ lack of time,^25^–^26^,^31^ and low patient demand for information^23^,^25^ as potential barriers, particularly in the preconception period.^20^,^23^,^25^,^32^ Better understanding the nature of these gaps may help determine how to best educate health care providers and build capacity. Overall, investing in reproductive and infant health can decrease poor birth and child outcomes and subsequently lower costs to health care, education, the justice system, non-profit organizations, and all levels of government.^33^–^35^

This article reports on a needs assessment targeting reproductive nurses and physicians who are in a position to address environmental health.^1^ The primary purpose was to explore the beliefs and knowledge of nurses and physicians about the impact of environmental toxicants on maternal and infant health, as well as to describe current practice and needs related to addressing EHIs. Questions guiding this needs assessment included: 1) To what extent do nurses and physicians consider it important to incorporate EHIs into their practice? 2) To what extent do they currently do so? 3) What are their current levels of knowledge regarding EHIs? 4) What are their current levels of confidence discussing EHIs with patients? 5) What are the barriers to incorporating EHIs into their practice? 6) What modes of assistance would be most helpful to them incorporating EHIs into their practice in the future?

## Methods

This project was approved by the University of Saskatchewan and Regina Qu’Appelle Health Region Research Ethics Boards. Ethics/operational approval was also obtained from each health region or authority in Saskatchewan.

### Participants

Eligible participants included nurses and physicians throughout Saskatchewan who were involved with caring for individuals contemplating pregnancy, pregnant women, and/or families with infants. The original needs assessment included a small number of midwives and other health support providers (e.g., dieticians, home visitors), but due to the low number of participants from these groups inferential statistical comparisons could not be made. Therefore, only nurses and physicians are the focus of this article. Invitations to complete the online survey were distributed via email through key health region contacts of the Saskatchewan Prevention Institute.^2^ These key facilitators were asked to distribute first the invitation to voluntarily and anonymously participate in the study and then a thank you or reminder to their contacts (colleagues, members of professional group email lists), who fit the participant criteria. The sample was one of convenience (i.e., non-random) due to limited resources and the nature of participant contacts.

Ninety-nine nurses (75 primary care/registered/public health/community nurses; 16 no specification of specialization; *and* 8 other, e.g., prenatal or labour and delivery nurses and directors/managers/case managers) and thirty-six physicians (29 generalists, e.g., family medicine; 7 specialists, e.g., maternal/neonatal/OBGYN, internal medicine, and public health/prevention) for a total of 135 (99 + 36)^3^ participants completed a questionnaire. Participants represented all health regions and authorities responsible for health services in the province.

### Questionnaire

The questionnaire was developed by consulting the literature on environmental health information in reproductive health care practice, utilizing aspects of validated and/or published questionnaires, identifying gaps in previous research designs, incorporating feedback from a Maternal and Infant Environmental Health Needs Assessment Advisory Committee organized by the Prevention Institute, and pilot testing. The questionnaire consisted of demographic questions (e.g., profession, region of practice), followed by quantitative and open-ended questions to assess the opinions, knowledge, practices, resources, and needs of respondents related to EHIs. The specific EHIs were selected based on what was presented within the literature (e.g., Government of Saskatchewan).^37^ Using a five-point scale, participants rated the importance of exploring EHIs during each reproductive time period (preconception, prenatal, and infant care), and the importance of discussing specific toxicants (Table 2) during patient care. To assess actual practice, participants rated the frequency with which they raised EHIs during each reproductive time period and how frequently they discussed specific toxicants with a patient in the last year. Participants also reported their level of knowledge regarding the impact of specific toxicants on reproductive health, and their confidence level discussing EHIs with patients. Additionally, participants identified the main barriers (see Table 3 in Appendix A) to raising EHIs with patients. Participants indicated how useful it would be to receive more information to help them address EHIs with patients, and the helpfulness of specific modes of assistance (Table 4). Finally, using text boxes, participants described additional barriers they experience to raising EHIs, and other resources or opportunities that would increase their capacity to do so.

## Results

Participants considered it somewhat important to explore EHIs in their practice and reported that they do so with moderate frequency for each time period (Table 1). Variations in responses regarding importance and actual practice across reproductive time periods were investigated via 2 one-way ANOVAs, both of which were significant at *p* < .001, *η_p_^2^* = .22 and *p* < .001, *η_p_^2^* = .31, respectively.^4^ Paired samples t-tests revealed that infant care was considered the most important time period in which to raise these issues and was also the time period in which they were most frequently discussed (followed by prenatal and then preconception care).^5^ There was no difference between physicians and nurses across time periods regarding importance of exploring EHIs, and no difference in actually exploring EHIs during the prenatal and infant time periods. However, nurses (*M* = 3.55, *SD* = 1.68) were significantly more likely than physicians (*M* = 3.00, *SD* = 1.00) to report exploring EHIs with patients during the preconception time period (*p* > 0.05, *d* = 0.37).

Both physicians (*M* = 2.33; *SD* = .84) and nurses (*M* = 2.28; *SD* = .89) reported low confidence in discussing specific EHIs with patients, with no group differences. While each group had somewhat high ratings for perceived importance of discussing EHIs with patients, nurses perceived some specific EHIs as significantly more important to discuss compared to physicians (Table 2).

While participants overall reported low knowledge levels regarding the impact of specific EHIs and frequency of discussing them (excluding tobacco smoke), there were some slight differences between physicians and nurses. In particular, physicians reported having significantly more knowledge on the impact of radon, radiation, and asbestos on infant health (see Table 3 in Appendix A). They also reported having more knowledge on the impact of mercury, lead, pesticides, radon, radiation, and asbestos on reproductive health. Additionally, while physicians were significantly more likely to have discussed mold, lead, radiation, and general occupational exposures with patients, nurses were more likely to have discussed bisphenol A and flame retardants (Table 4).

The relationship between perceived importance, knowledge, and frequency of discussing EHIs in practice was explored in more depth. Both knowledge (*r* = .674, *p* < .001) and importance (*r* = .211, *p* = .018) were significantly positively correlated with frequency of discussing EHIs. Knowledge was not significantly correlated with importance. In a hierarchical regression, knowledge, entered first, significantly accounted for 45% of the variance in reported frequency of discussing EHIs, [*R*^2^ = .45, *F*(1, 96) = 79.86, *p* < .001]. When perceived importance was added in the second block, it significantly accounted for only 2.3% more variance than did knowledge alone [*R*^2^_change_ = .023, *F*_change_(1,95) = 4.13, *p* = .045].

Moreover, “lack of knowledge” was the second most frequently selected barrier to discussing EHIs, following only “time pressure” (Table 5). Participants commented on the importance of knowledge: “We have very little education in most of the areas discussed” (public health nurse); “I also feel ill-equipped to provide detailed responses to several of these potential environmental pollutants” (physician).

Overall, participants reported that more information about the impact of EHIs would be helpful for each care period.^6^ Variations in responses across reproductive time periods were investigated via a one-way ANOVA, which was significant [*F*(2, 198) = 12.05, *p* < .001, *η_p_^2^* = .11]. In a pattern similar to perceived importance and frequency of discussing EHIs, paired samples t-tests showed that participants considered it most helpful to receive EHI information pertaining to infant care, followed by prenatal and then preconception care.^7^

Receiving quality online informational resources, lists of patient-targeted online resources, clinical practice tools, and booklets for patients were perceived as most helpful, while in-clinic educational posters were reported as least helpful (Table 6). Many participants elaborated qualitatively that informational resources would increase their capacity to address EHIs, particularly online resources. Specifically, the potential of a toxicant manual/toolkit for professional reference was identified by several respondents: “An on-line environmental health manual for professionals would be nice to be able to access.” (public health nurse). Training opportunities for health care providers were also identified as potentially helpful, particularly training sessions and webinars: “Webinars, training sessions and environmental emails/manuals/online resource sites [are] very, very helpful” (public health nurse); “training and teaching” (physician).

## Discussion

Three main points can be garnered from the results of this study. First, EHIs appear to be perceived differently depending on the type of reproductive care being provided. Raising EHIs was rated as most important when providing infant care, and least important in preconception care; this same pattern emerged regarding actual practice and perceived helpfulness of more information. Nurses and physicians may be less aware of the potential harms of environmental toxicants prior to conception, and/or feel less able to address issues during this period. Previous work has suggested that health care providers and patients undervalue preconception care, and factors such as unplanned pregnancies and irregular health care may make addressing EHIs during preconception difficult.^19^,^20^,^23^,^25^,^31^, This may be an important gap to address in Canadian medical education, given that preconception exposure to toxicants can negatively impact the reproductive health of women and adversely affect their children.^17^

Second, although participants believed exploring EHIs is generally important, our findings suggest they are not routinely addressed in practice which corresponds to previous findings in the United States.^21^,^25^,^27^,^28^ Our results suggest that lack of knowledge (rather than perceived importance) is a significant barrier to effectively addressing EHIs, as it accounted for almost half of the variance in frequency of discussing EHIs. This finding coincides with research in other geographic contexts suggesting health care professionals do not feel well-equipped to adequately inform their patients about environmental health concerns.^9^,^21^,^26^,^29^,^38^–^40^

Third, participants indicated that it would be helpful to receive additional information, resources, and training on EHIs. All modes of assistance were perceived as helpful, but online informational resources for both health care providers and patients were considered particularly beneficial. Participants viewed health care providers as important points of access to environmental health information for patients, and reported that increased access to quality information would increase their capacity to understand, address, and educate patients on EHIs. The findings from this needs assessment suggest more information, resources, and education on EHIs are desired by physicians and nurses. Ultimately, this could increase both their knowledge levels of EHIs and the frequency with which they address them with patients.

### Limitations

Important limitations to note are that the sample was not random and may not be wholly representative. Although efforts were made to reach all physicians and nurses who work in preconception, prenatal, and infant health in Saskatchewan, the required resources to achieve this were not available. However, the sample included a wide range of healthcare professionals from every health region in the province, suggesting that some degree of coverage was obtained.

Another limitation pertains to the generalizability of the needs assessment materials and findings to other Canadian jurisdictions. Provinces may vary in terms of their policies and practices regarding EHIs, and may differ with respect to the types of EHIs that are of most concern. Further, it was beyond the scope of this needs assessment to examine issues related to the underlying causes of patient exposure to EHIs, a serious public health issue which healthcare provider education alone cannot ameliorate.

Despite these limitations, this needs assessment adds to the limited amount of information regarding how EHIs are addressed in reproductive care in Canada.

### Conclusions and recommendations

The initial intent of this needs assessment was to explore the beliefs, knowledge, current practice, and needs of nurses and physicians related to addressing EHIs within the scope of maternal and infant healthcare. Based on the findings, some continuing education considerations and recommendations have been developed. While many resources regarding the impact of environmental toxicants on reproductive and infant health are available (e.g., Society of Obstetricians and Gynaecologists of Canada; SOGC, 2010), results from our needs assessment suggest that the uptake and application of them may not be consistent across healthcare professionals. There may be a need to adjust training programs and policies regarding EHIs and reproductive and infant health care practices, including placing greater emphasis on the importance of assessing and understanding the impact of EHIs, in general, and during the preconception care period, in particular. Further, it may be necessary to develop new resources or update existing ones to ensure that they are of high quality, easy-to-access, and available online for both healthcare professionals and patients.

## Figures and Tables

**Table 1:** Mean responses by time period

	Preconception		Prenatal		Infant	
	*n*	*M (SD*)	*n*	*M* (*SD*)	*n*	*M (SD)*
Mean perceived importance of exploring EHIs in general*	118	4.06 (1.10)	120	4.38 (1.05)	121	4.46 (1.06)
Mean frequency of raising EHIs in general**	115	2.86 (1.12)	117	3.47 (1.04)	120	3.94 (0.98)

* Scale: 1- *very unimportant*; 2- *unimportant*; 3- *neutral*; 4- *important*; 5- *very important*

** Scale: 1- *never*; 2- *rarely*; 3- *sometimes*; 4- *often*; 5- *always*

**Table 2:** Group differences on importance discussing specific EHIs

	Importance of discussing specific toxicants*		
Toxicant	Physicians (n = 35)	Nurses (n = 87)	Group differences
	*M*(*SD*)	*M*(*SD*)	*p, d*
**Mold**	3.63 (0.69)	4.08 (0.96)	*p* = 0.01 *d* = 0.51
**Mercury**	3.60 (0.81)	3.99 (0.99)	*p* = 0.04 *d* = 0.41
**Lead**	3.74 (0.74)	4.02 (1.02)	*ns*
**Pesticides**	3.69 (0.76)	4.03 (0.99)	*ns*
**Tobacco smoke**	4.57 (0.61)	4.46 (0.97)	*ns*
**Bisphenol A** (**BPA)**	3.26 (0.92)	3.94 (0.99)	*p* = 0.001 *d* = 0.70
**Radon**	3.26 (0.87)	3.72 (1.00)	*p* = 0.02 *d* = 0.48
**Radiation**	3.46 (0.85)	3.86 (1.05)	*p* = 0.05 *d* = 0.40
**Asbestos**	3.49 (0.89)	3.82 (1.08)	*ns*
**Outdoor air quality**	3.37 (0.73)	3.87 (0.97)	*p* = 0.01 *d* = 0.55
**Indoor air quality**	3.77 (0.88)	4.14 (0.97)	*ns*
**Water quality**	3.71 (0.75)	4.28 (0.96)	*p* = 0.002 *d* = 0.63
**Occupational exposures**	3.74 (0.74)	4.00 (0.92)	*ns*
**Food toxicants**	3.51 (0.74)	4.01 (0.99)	*p* = 0.01 *d* = 0.54
**Flame retardants**	3.06 (0.73)	3.87 (0.99)	*p* < 0.001 *d* = 0.88
**Household product toxicants**	3.60 (0.85)	4.01 (0.98)	*p* = 0.03 *d* = 0.43

* Scale: 1- very unimportant; 2- unimportant; 3- neither important nor unimportant; 4- important; 5- very important

**Table 4:** Mean frequency of discussing specific toxicants and group differences between physicians and nurses

	Frequency estimates of discussing specific toxicants in last year*		
Toxicant	Physicians (n = 30)	Nurses (n = 70)	Group differences
	*M*(*SD*)	*M*(*SD*)	*p, d*
**Mold**	3.03 (0.85)	2.39 (0.99)	*p* = 0.002 *d* = −0.67
**Mercury**	2.13 (1.22)	1.79 (1.05)	*ns*
**Lead**	1.93 (1.02)	1.48 (0.81)	*p* = 0.04 *d* = − 0.51
**Pesticides**	2.10 (1.06)	2.09 (1.08)	*ns*
**Tobacco smoke**	4.57 (0.68)	4.57 (0.86)	*ns*
**Bisphenol A (BPA)**	1.37 (0.72)	1.97 (1.17)	*p* = 0.002 *d* = 0.57
**Radon**	1.23 (0.68)	1.26 (0.65)	*ns*
**Radiation**	2.13 (1.33)	1.42 (0.82)	*p* = 0.01 *d* = −0.71
**Asbestos**	1.73 (1.02)	1.44 (0.88)	*ns*
**Outdoor air quality**	3.10 (1.30)	3.00 (1.20)	*ns*
**Indoor air quality**	3.17 (1.21)	3.34 (1.40)	*ns*
**Water quality**	2.77 (1.43)	3.23 (1.34)	*ns*
**Occupational exposures**	3.13 (1.36)	2.23 (1.23)	*p* = 0.002 *d* = −0.71
**Food toxicants**	2.30 (1.29)	2.38 (1.14)	*ns*
**Flame retardants**	1.17 (0.75)	1.54 (0.92)	*p* = 0.04 *d* = 0.42
**Household product toxicants**	2.33 (1.37)	2.39 (1.20)	*ns*

* Scale: 1- never; 2- once; 3- a few times; 4- once a month; 5- once a week

**Table 5:** Barriers to raising environmental health issues or taking environmental health history: Frequency of endorsement

Barrier	N	%
Time pressure	84	62.2
Lack of knowledge	76	56.3
Importance in relation to other issues	74	54.8
Patient has little control of issues	55	40.7
Lack of capacity to treat of refer	52	38.5
Patient is not interested	50	37.0
Difficulty communicating risk to patients	22	16.3
Limited/contradictory research on impact of exposure	16	11.9
Concern about patient reaction	6	4.4

**Table 6:** Mean perceived helpfulness of different modes of assistance

	Total (n = 101)
	*M (SD)*
Online information resources for care providers	4.31 (.72)
List of patient-targeted online resources	4.01 (.87)
Clinical practice tools	3.90 (.78)
Booklets or pamphlets to provide to patients	3.86 (.93)
Webinars	3.85 (.89)
Environmental health manual for care providers	3.81 (.90)
Including EHH on prenatal forms	3.77 (.86)
In-person training sessions	3.73 (.87)
In-clinic educational posters	3.70 (.88)

Scale: 1- very unhelpful; 2- unhelpful; 3- neutral; 4- helpful; 5- very helpful

## Footnotes

1 This needs assessment was commissioned by the Saskatchewan Prevention Institute (a nonprofit organization with a mission to reduce the occurrence of disabling conditions in children) in order to explore needs that could be addressed through provincial capacity-building efforts.

2 A comprehensive list of contact information for health care providers in the province was not available.

3 Due to the method of recruitment (i.e., convenience/snowball sampling with key facilitators distributing invitations to their contacts), the number of surveys distributed and response rate could not be calculated. According to Saskatchewan Prevention Institute records, there are currently approximately 965 physicians who provide prenatal care in Saskatchewan. The Saskatchewan Registered Nurses Association reported that the number of Registered Nurses licensed in 2016 in Saskatchewan is 11, 491.^36^

4 *F**_Importance_*(2, 218) = 30.60 and *F**_Frequency_*(2, 182) = 41.12

5 All p-values < .01.

6 *Mpreconception* = 3.65 (*SD* = 1.10); *Mprenatal* = 3.89 (*SD* = 1.10); *Minfant* = 4.08 (*SD* = 0.95)

7 All p-values < .01

## Appendix A

**Table 3:** Comparing knowledge levels among physicians and nurses of effects of specific toxicants

	Knowledge levels specific toxicants infant health*			Knowledge levels specific toxicants reproductive health*		
Toxicant	Physicians (n = 31)	Nurses (n = 78)	Group differences	Physicians (n = 31)	Nurses (n = 78)	Group differences
	*M*(*SD*)	*M*(*SD*)	*p, d*	*M*(*SD*)	*M*(*SD*)	*p, d*
**Mold**	2.48 (0.96)	2.26 (0.80)	*ns*	2.10 (0.65)	1.90 (0.78)	*ns*
**Mercury**	2.45 (1.00)	2.09 (0.79)	*ns*	2.39 (0.76)	1.96 (0.76)	*p* = 0.01 *d* = −0.57
**Lead**	2.52 (0.96)	2.15 (0.77)	*ns*	2.35 (0.84)	1.95 (0.70)	*p* = 0.01 *d* = −0.54
**Pesticides**	2.32 (0.95)	2.14 (0.80)	*ns*	2.42 (0.72)	2.04 (0.76)	*p* = 0.02 *d* = −0.51
**Tobacco smoke**	3.53 (0.94)	3.40 (0.80)	*ns*	3.61 (0.72)	3.27 (0.90)	*ns*
**Bisphenol A** (**BPA)**	2.00 (1.00)	2.12 (0.87)	*ns*	1.87 (0.92)	1.90 (0.74)	*ns*
**Radon**	2.03 (1.02)	1.54 (0.68)	*p* < 0.001 *d* = −0.62	1.94 (0.81)	1.58 (0.64)	*p* = 0.02 *d* = −0.52
**Radiation**	2.55 (1.03)	1.99 (0.71)	*p* < 0.001 *d* = −0.69	2.81 (1.05)	1.97 (0.72)	*p* < 0.001 *d* = −1.02
**Asbestos**	2.32 (0.98)	1.92 (0.68)	*p* = 0.02 *d* = −0.52	2.23 (0.92)	1.86 (0.70)	*p* = 0.03 *d* = −0.48
**Outdoor air quality**	2.65 (0.99)	2.47 (0.88)	*ns*	2.39 (0.84)	2.28 (0.90)	*ns*
**Indoor air quality**	2.71 (0.94)	2.59 (0.86)	*ns*	2.48 (0.93)	2.40 (0.93)	*ns*
**Water quality**	2.74 (1.03)	2.69 (0.87)	*ns*	2.55 (0.93)	2.57 (0.95)	*ns*
**Occupational exposures**	2.16 (0.93)	2.08 (0.82)	*ns*	2.42 (0.67)	2.31 (0.81)	*ns*
**Food toxicants**	2.40 (0.97)	2.17 (0.79)	*ns*	2.32 (0.75)	2.18 (0.73)	*ns*
**Flame retardants**	1.77 (0.85)	1.78 (0.82)	*ns*	1.68 (0.65)	1.72 (0.72)	*ns*
**Household product toxicants**	2.39 (0.96)	2.14 (0.86)	*ns*	2.06 (0.77)	2.09 (0.79)	*ns*

* Scale: 1- *no knowledge*; 2- *basic awareness*; 3- *intermediate*; 4- *advanced*; 5- *expert*

## References

[1] Stillerman KP, Mattison DR, Giudice LC, Woodruff TJ (2008). Environmental exposures and adverse pregnancy outcomes: a review of the science. Reprod Sci.

[2] Cooper K, Marshall L, Vanderlinden L, Ursitti F Early Exposures to Hazardous Pollutants/Chemicals and Associations with Chronic Disease - a scoping review [Internet]. Canadian Environmental Law Association and Ontario College of Family Physicians 2011.

[3] Munoz-Quezada MT, Lucero BA, Barr DB, Steenland K, Levy K, Ryan PB (2013). Neurodevelopmental effects in children associated with exposure to organophosphate pesticides: a systematic review. Neurotoxicology.

[4] Rochester JR (2013). Bisphenol A and human health: a review of the literature. Reprod Toxicol.

[5] Crain DA, Janssen SJ, Edwards TM, Heindel J, Ho SM, Hunt P (2008). Female reproductive disorders: the roles of endocrinedisrupting compounds and developmental timing. Fertil Steril.

[6] Chen A, Yolton K, Rauch SA, Webster GM, Hornung R, Sjodin A (2014). Prenatal Polybrominated Diphenyl Ether Exposures and Neurodevelopment in U.S. Children through 5 Years of Age: The HOME Study. Environ Health Perspect.

[7] Wigle DT, Arbuckle TE, Turner MC, Berube A, Yang Q, Liu S (2008). Epidemiologic evidence of relationships between reproductive and child health outcomes and environmental chemical contaminants. J Toxicol Environ Health B Crit Rev.

[8] Jedrychowski W, Perera F, Maugeri U, Miller RL, Rembiasz M, Flak E (2011). Intrauterine exposure to lead may enhance sensitization to common inhalant allergens in early childhood: A prospective prebirth cohort study. Environ Res.

[9] Trasande L, Ziebold C, Schiff JS, Wallinga D, McGovern P, Oberg CN (2008). The role of the environment in pediatric practice in Minnesota. Minnesota Medicine.

[10] Diamanti-Kandarakis E, Bourguignon JP, Giudice LC, Hauser R, Prins GS, Soto AM (2009). Endocrinedisrupting chemicals: An Endocrine Society scientific statement. Endocrine Reviews.

[11] Wilson D (2011). Genetic considerations for a woman’s pre-conception evaluation. Journal of Obstetrics and Gynaecology Canada.

[12] Wong S, Ordean A, Kahan M (2011). Substance use in pregnancy. Journal of Obstetrics and Gynaecology Canada.

[13] Zoeller RT, Brown TR, Doan LL, Gore AC, Skakkebaek NE, Soto AM (2012). Endocrine-disrupting chemicals and public health protection: a statement of principles from The Endocrine Society. Endocrinology.

[14] University of California San Francisco Program on Reproductive Health and the Environment. Professional statements database [Internet].

[15] Society of Obstetricians and Gynaecologists of Canada Clinical practice guidelines [Internet].

[16] Association of Reproductive Health Professionals (ARHP) (2010). Environmental impacts on reproductive health [Internet]. Association of Reproductive Health Professionals Clinical Proceedings.

[17] The American College of Obstetricians and Gynecologists Committee on Health Care for Undeserved Women (ACOG Committee) (2013). Exposure to toxic environmental agents. American Society for Reproductive Medicine Pages.

[18] Crighton E, Abelsohn A, Blake J, Enders J, Kilroy K, Lanphear B, Smith G (2016). Beyond alcohol and tobacco smoke: Are we doing enough to reduce fetal toxicant exposure?. J Obstet Gynaecol Can.

[19] Riskin-Mashiah S, Auslander R (2007). Preconception care - when and what: The attitude of Israeli gynaecologists to preconception counseling. Arch Gynecol Obstet.

[20] Delvoye P, Guillaume C, Collard S, Nardella T, Hannecart V, Mauroy MC (2009). Preconception health promotion: analysis of means and constraints. Eur J Contracept Reprod Health Care.

[21] Stotland NE, Sutton P, Trowbridge J, Atchley DS, Conry JA, Trasande L (2014). Counseling Patients on Preventing Prenatal Environmental Exposures - A Mixed-Methods Study of Obstetricians. PLoS One.

[22] Laferriere KA, Crighton EJ, Baxter J, Lemyre L, Masuda JR, Ursitti F (2016). Examining inequities in children’s environmental health: Results of a survey on the risk perceptions and protective actions of new mothers. Journal of Risk Research.

[23] Heyes T, Long S, Mathers N (2004). Preconception care: Practice and beliefs of primary care workers. Family Practice.

[24] Poppelaars FAM, Cornel MC, Kate LP (2004). Current practice and future interest of GPs and prospective parents in pre-conception care in The Netherlands. Fam Prac.

[25] Morgan MA, Hawks D, Zinberg S, Schulkin J (2006). What obstetrician-gynecologists think of preconception care. Matern Child Health J.

[26] van Heesch PN, de Weerd S, Kotey S, Steegers EA (2006). Dutch community midwives’ views on preconception care. Midwifery.

[27] Trasande L (2006a). Pediatrician attitudes, clinical activities, and knowledge of environmental health in Wisconsin. Wiscon Med J.

[28] Trasande L (2006b). The environment in pediatric practice: A study of New York pediatricians’ attitudes, beliefs, and practices towards children’s environmental health. J Urb Health: Bullet NY Acad Med.

[29] Braspenning S, Haagdorens M, Blaumeiser B, Jacquemyn Y, Mortier G (2013). Preconceptional care: A systematic review of the current situation and recommendations for the future. Facts Views Vis Obygn.

[30] Trasande L (2014). The environment and children’s health care in Northwest China. BMC Ped.

[31] Curtis M, Abelman S, Schulkin J, Williams JL, Fassett EM (2006). Do we practice what we preach? A review of actual clinical practice with regards to preconception care guidelines. Matern Child Health J.

[32] Sharma S, Ashley JM, Hodgson A, Nisker J (2014). Views of pregnant women and clinicians regarding discussion of exposure to phthalate plasticizers. Repro Health.

[33] Ministry of Health Promotion Reproductive Health Guidance Document [Internet].

[34] Muir T, Zegarac M (2001). Societal costs of exposure to toxic substances: economic and health costs of four case studies that are candidates for environmental causation. Environ Health Perspect.

[35] Di Renzo GC (2015). International Federation of Gynecology and Obstetrics opinion on reproductive health impacts of exposure to toxic environmental chemicals. International Journal of Gynecology and Obstetrics.

[36] Saskatchewan Registered Nurses Association Safe, Trusted Care Through Collaboration [Internet].

[37] Government of Saskatchewan (n.d). Prevention and protection: Environmental health [Internet].

[38] Tough SC, Clarke M, Hicks M, Clarren S (2005). Attitudes and approaches of Canadian providers to preconception counselling and the prevention of fetal alcohol spectrum disorders. J FAS Internat.

[39] Gaytant MA, Cikot RJ, Braspenning JC (1998). Preconception counseling in family practice: A survey of 100 family physicians. Ned Tijdschr Geneeskd.

[40] Schrander-Stumpel C (1999). Preconception care: Challenge of the new millennium?. Am J Med Gen.

